# Improved Femoroacetabular Range of Motion in Runners Following the Spencer Technique for the Hip: A Secondary Analysis of a Randomized Controlled Trial

**DOI:** 10.7759/cureus.87908

**Published:** 2025-07-14

**Authors:** Zachary J Buchman, Benjamin M Vinarski, Jason S DeFrancisis, Dante DiSilvestro, Daniel P Oar, Jack T Carter, Jesse T O'Rorke, David Boesler, James Toldi, Rebecca E Steiner

**Affiliations:** 1 Physical Medicine and Rehabilitation, Lake Erie College of Osteopathic Medicine, Bradenton, USA; 2 Orthopedic Surgery, Lake Erie College of Osteopathic Medicine, Bradenton, USA; 3 Medicine, Lake Erie College of Osteopathic Medicine, Bradenton, USA; 4 Internal Medicine, Lee Health, Fort Myers, USA; 5 Osteopathic Medicine, Lake Erie College of Osteopathic Medicine, Bradenton, USA; 6 Sports Medicine, Lincoln Memorial University DeBusk College of Osteopathic Medicine, Orange Park, USA

**Keywords:** athletic performance, femoroacetabular joint, musculoskeletal medicine, omt in sport, orthopedic sports medicine, osteopathic manipulative medicine, osteopathic manipulative treatment (omt), physical medicine and rehabilitation, spencer technique, spencer technique of the hip

## Abstract

Introduction: The Spencer technique of the hip is an osteopathic manipulative technique used to treat somatic dysfunction and increase hip range of motion (ROM), including flexion, extension, internal rotation, external rotation, abduction, and adduction. The application of this osteopathic manipulative technique in runners versus non-runners to assess potential differences in improvements of femoroacetabular ROM has not been clinically investigated in existing literature.

Objective: The goal of this research was to determine if the application of the Spencer technique of the hip improves femoroacetabular ROM in runners compared to non-runners.

Materials and methods: In this retrospective analysis of results from a randomized controlled trial, the effects of the Spencer technique on the hip were investigated in nine runners and eleven non-runners (n = 20). The Spencer technique of the hip was performed on all participants twice per week for four weeks. Participants were retrospectively categorized into either “runners” or “non-runners” groups based on self-reported exercise habits during the treatment period. “Runners” self-reported running a ten-minute mile, or faster, on at least two days per week for at least two of the four weeks investigated, whereas those in the “non-runners” group did not report exercising to this threshold. Before and after the four-week treatment period, goniometer ROM measurements were recorded. Using averaged bilateral data, the average percent change over the four weeks for each motion was compared between the two groups using a two-sample, two-tailed t-test.

Results: One significant difference was found comparing the average percentage change of hip internal rotation over four weeks of treatment in runners (+31.7% ± 29.8) compared to non-runners (-8.6% ± 17.8) for a difference of 40.3% (95% CI: (15.40, 65.20), p = 0.001). The average percent change over the four weeks for all other motions (flexion, extension, external rotation, abduction, and adduction) when compared between groups demonstrated no significant difference (p > 0.05).

Conclusions: The results obtained in this study indicate that exercise programs that rely more on aerobic training may play a significant role in the impact of the Spencer technique therapy on the femoroacetabular joint, specifically in internal rotation. These findings are limited by a small sample size (n = 20) and restricted participant demographics (strictly medical students aged 21-30), creating the need for samples of larger quantity and greater diversity in future studies of this topic. These findings may help osteopathic physicians more effectively utilize the Spencer technique when treating their diverse patient populations with varying aerobic exercise habits.

## Introduction

Osteopathic manipulative treatment (OMT) has long been utilized to improve musculoskeletal function and overall mobility in various populations, from athletes to sedentary individuals [1-3]. The Spencer technique is an OMT protocol that can be employed to enhance joint mobility and address myofascial restrictions in either the glenohumeral or femoroacetabular joints, depending on its application [4]. Although research has been completed investigating the Spencer technique of the shoulder in both athletic and non-athletic populations, the application of the Spencer technique of the hip and its potential benefits in specific athletic populations, such as runners, remains understudied [2,3]. Given the repetitive motion and postural demands placed on runners, it can be hypothesized that their musculoskeletal adaptations and restrictions may differ significantly from those of non-runners, necessitating further investigation into the impact of the Spencer technique on the hip in varying patient populations [5].

Previous research has been conducted to determine the impact that OMT has on runners [6]. However, there is limited reference and a lack of data in the existing literature specifically regarding the use of the Spencer technique of the hip and its effects on runners' femoroacetabular range of motion (ROM). The Spencer technique is an articulatory/springing technique performed by bringing the joint to its restrictive barrier and applying rhythmic, repetitive, direct, and indirect movements through the hip's ROM [4]. This is done in eight consecutive steps following the order of flexion, extension, circumduction with traction, circumduction with compression, internal rotation, external rotation, abduction, and adduction [4]. Existing literature has failed to demonstrate a positive relationship between the Spencer technique of the hip and improved ROM, in that one study investigating this relationship reported a possible negative relationship between the technique and ROM compared to control over four weeks [7]. In contrast to the Spencer technique of the hip, the Spencer technique of the shoulder has received much more attention and has, in many studies, proven to be a valuable option in improving shoulder ROM [2,8-10]. In Curcio's study, it was shown that overhand baseball throwing can negatively impact glenohumeral joint ROM and that the Spencer technique of the shoulder can be effectively used to counteract this undesirable change in collegiate baseball players [2]. Further, Phansopkar's study demonstrated that the Spencer technique of the shoulder can positively impact ROM in a patient with adhesive capsulitis [10]. This ROM improvement occurred in only three weeks, one week shorter than the length of treatment applied in the presented study [10].

In addition to the functionality of the femoroacetabular joint in hip ROM, lower extremity musculature plays an essential role in joint function as well [11]. In particular, the hamstrings play a vital role in hip mobility, and hypertonicity can result in severely restricted joint motion [11]. Taking this anatomical consideration into mind, studies that examine the role of OMT in addressing hamstring muscle hypertonicity have implications for this study as well. Utilizing motions similar to those of the Spencer technique of the hip, hamstring muscle energy has been shown to decrease muscle tension and increase hip ROM [12]. Although the Spencer technique mainly focuses on the joint structure itself, the promising results of similar OMT techniques suggest that it may also alleviate restrictions imposed by the surrounding musculature on joint mobility [12].

Despite the growing body of evidence supporting OMT, disagreement exists regarding its efficacy and the degree to which manual therapy contributes to measurable performance improvements in athletes [2,13]. To address this debate, this study aims to examine whether runners derive distinct, measurable benefits from the Spencer technique of the hip compared to non-runners also treated with the same approach. By examining differences in ROM measurements between runners and non-runners, both treated with the Spencer technique of the hip, this study aims to fill a gap in the existing literature and help osteopathic physicians build a more standardized treatment approach to ensure optimal clinical outcomes in diverse patient populations with varying levels of physical activity. This study hypothesizes that those who engage in regular physical activity, such as running, will have a greater response to the Spencer technique of the hip compared to sedentary individuals due to differences in myofascial adaptability, efficiency, and baseline musculoskeletal health.

## Materials and methods

The ROM measurement data analyzed in this manuscript were collected during a previous study by O'Rorke et al. to evaluate the impact of the Spencer technique on femoroacetabular ROM and femoroacetabular mobility [7]. During this process, the Lake Erie College of Osteopathic Medicine Institutional Review Board approved this study (approval number: 31-085), and a post-hoc clinical trial registry was made through ClinicalTrials.gov, being assigned the number NCT06644989 [7]. Study funding was provided through Lake Erie College of Osteopathic Medicine Consortium of Academic Excellence Grant #J2023.23, and $2550.00 was appropriated to finance the project [7]. Informed consent was obtained from all participants, which occurred before the start of the study and after the initial screening requirements [7]. Original informed consent was completed on a paper form explaining the summary of information, the purpose of the study, inclusion and exclusion criteria (Tables 1-2), procedures, potential risks, potential benefits, confidentiality, voluntary participation, withdrawal from participation, involuntary withdrawal, and an opportunity for prospective participants to ask questions [7]. This informed consent document contained a clause granting permission for secondary research to be conducted using de-identified data [7]. Signatures of all participants were obtained on physical informed consent forms, and participants were compensated monetarily using funds appropriated by the provided grant after completion of the study [7].

**Table 1:** Inclusion criteria for participant recruitment and enrollment All of the inclusionary criteria were used in participant recruitment and enrollment. To be successfully enrolled, the potential participant had to agree to complete, or be in compliance with, all of the inclusionary criteria. This information was originally published in the primary research and may also be found in O'Rorke et al. [7]. LECOM: Lake Erie College of Osteopathic Medicine

Inclusion criteria	Must meet all to be included in the study
1	Be a current first- or second-year medical student at LECOM, Bradenton, between the ages of 21 and 30
2	Attend all measurement sessions
3	Attend 100% of treatment sessions
4	Respond to 100% of exercise surveys

**Table 2:** Exclusion criteria used in participant recruitment and enrollment All of the exclusionary criteria were used in participant recruitment and enrollment. To be successfully enrolled, the potential participant had to agree to complete, or be in compliance with, none of the exclusion criteria. This information was originally published in the primary research and may also be found in O'Rorke et al. [7].

Exclusion criteria	None may be met to be included in the study
1	If they cannot sign an informed consent
2	If they are pregnant or plan to become pregnant
3	If they have a past medical history which increases the risk of adverse reactions including, but not limited to, any of the following conditions: skin disorders or open wounds precluding skin contact, neurological symptoms (i.e. numbness, tingling, weakness), adhesive capsulitis of the hip joint, osteoarthritis of the lower extremity, rheumatoid arthritis, gout, iliotibial band syndrome, Legg-Calve-Perthes disease, slipped capital femoral epiphysis, hip dysplasia, avascular necrosis of the hip, chronic hip bursitis, hip dislocation, osteoporosis/osteopenia, severe femoroacetabular impingement, immunosuppressive syndromes, radiation or chemotherapy within the past three years, congestive heart failure, Down syndrome, have been told by a physician or other medical professional that you should not participate in aerobic training activities, or have medication changes in the last month
4	If they have a past medical history which increases confounding variables to the study including, but not limited to, any of the following conditions: recent bone fracture, use of heel lift due to leg length discrepancy, recent lower extremity ligamentous sprain, recent lower extremity muscle strain, history of hip or knee arthroplasty, or one year history of any lower extremity surgery
5	Additionally, participants will be excluded if they report that they run beyond what would be considered a "recreational runner," that being 30 miles per week

To test the precision of the goniometer measurements prior to study onset, preliminary data were collected by each member of the research team, measuring all planes of motion on six standard individuals [7]. The measurements taken by each member of the research team for each motion, on each standard individual, were compiled to generate average ranges of motion and average standard deviations for each motion, results of which can be found in the results section of this manuscript [7]. Also, before the study's onset, all members of the research team who were medical students were assessed in their ability to provide treatment to study subjects. To ensure proficiency, ahead of treatment, all authors exhibited capability through a series of demonstrations of the Spencer technique, following the direction of the Atlas of Osteopathic Techniques [4,7]. An experienced osteopathic physician oversaw and confirmed their proficiency via two consecutive successful demonstrations. To ensure equal treatment was provided to participants of the treatment group, the Spencer technique was standardized and performed as demonstrated in Table 3 [4,7].

**Table 3:** Protocol for the Spencer technique applied to the hip in this study Each step was performed bilaterally and as adherent to the directions provided in the Atlas of Osteopathic Techniques as possible, with standardized procedures. Standardized procedures were required to be implemented as there is variation in terms of the acceptable number of thrusts or circumductions per stage. For all stages, the patient is in a supine position, and each step was done once per leg at each treatment session. This information was originally published in the primary research and may also be found in O'Rorke et al. [7]. ASIS: anterior superior iliac spine, ROM: range of motion

Stages	Steps
Stage 1: hip flexion	1) The physician stands next to the hip being treated
	2) The physician flexes the knee, carries the hip to the flexion-restrictive barrier, and applies an articulatory motion five times at the end ROM
Stage 2: hip extension	1) The physician stands next to the hip being treated
	2) The physician moves the patient's leg slightly off the table laterally, depresses at the distal thigh to the extension-restrictive barrier, and applies an articulatory motion five times at the end ROM
Stage 3: circumduction with compression	1) The physician stands next to the hip being treated
	2) The physician flexes the patient's hip (with the knee flexed) to the flexion-restriction barrier and adds slight to moderate compression
	3) The physician circumducts the patient’s hip through five small and enlarging circles (clockwise, then counterclockwise directions) while maintaining compression
Stage 4: circumduction with traction	1) The physician stands at the foot of the bed
	2) The physician extends the patient’s knee and grasps the ankle, adding slight to moderate traction
	3) The physician circumducts the patient’s hip through five small and enlarging circles (clockwise, then counterclockwise directions) while maintaining traction
Stage 5: internal rotation	1) The physician stands next to the hip being treated
	2) The physician flexes the patient's hip and knee, then brings the hip to its internal rotation-restrictive barrier
	3) While stabilizing the ipsilateral ASIS, an articulatory motion is applied five times at the end ROM
Stage 6: external rotation	1) The physician stands opposite the hip being treated
	2) The physician flexes the patient's hip and knee, then brings the hip to its external rotation-restrictive barrier
	3) While stabilizing the contralateral ASIS, an articulatory motion is applied five times at the end ROM
Stage 7: abduction	1) The physician stands next to the hip being treated
	2) The physician takes the patient’s straightened leg and brings it to its abduction-restrictive barrier
	3) The physician stabilizes the ipsilateral ASIS, and an articulatory motion is applied five times at the end ROM
Stage 8: adduction	1) The physician stands next to the hip being treated
	2) The patient’s leg is slightly elevated with the knee extended
	3) The physician takes the patient’s straightened leg and brings it to its adduction-restrictive barrier
	4) The physician stabilizes the ipsilateral ASIS, and an articulatory motion is applied five times at the end ROM

Participants in the study consisted of first- and second-year medical students who were recruited via email. Interested medical students responded to the email, and potential participants were screened according to the inclusion and exclusion criteria (Tables 1-2); those eligible to participate in the study were selected. Participant selection was done consecutively, and the 40 participants agreed and signed consent forms, officially enrolling them in the original study. Data from 20 of these participants, all of whom were in the "treatment group" mentioned in O'Rorke et al., were retrospectively analyzed for the secondary research presented in this manuscript [7]. The 20 participants from O'Rorke et al. not treated with the Spencer technique were excluded from the retrospective data analysis presented in this manuscript [7]. For this manuscript, the following methodology refers only to actions taken by the 20 participants whose data are analyzed in this manuscript. Any unique actions taken by participants from the original O'Rorke et al. manuscript, which did not produce data analyzed in this manuscript, have been omitted [7].

**Figure 1:**
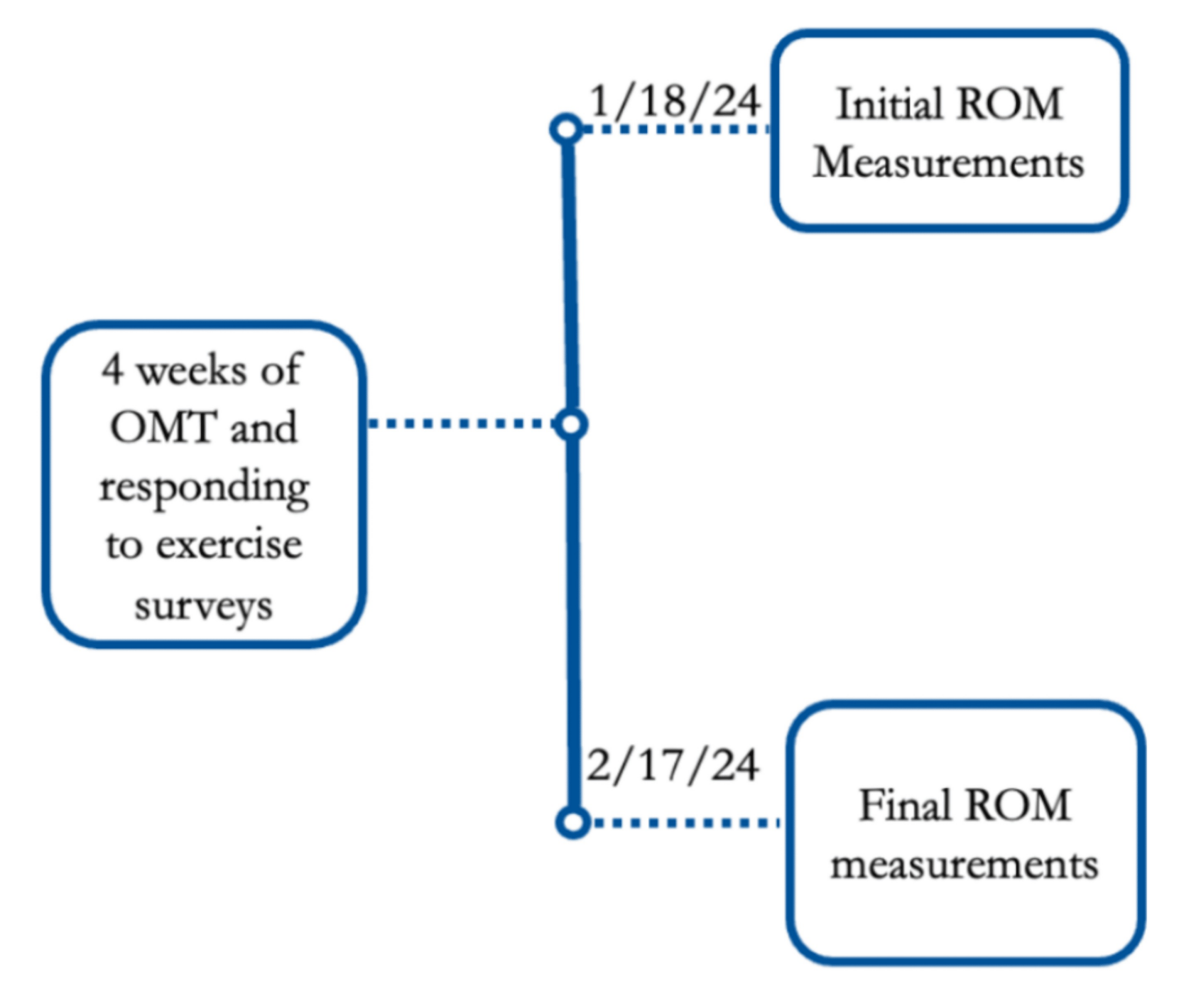
Timeline of the study The timeline of the study, including associated dates and events, was completed. OMT: osteopathic manipulative treatment, ROM: range of motion

After the participants were officially enrolled, all participants attended a session where their initial hip ROM measurements were taken by members of the research team using handheld goniometers. These included flexion, extension, internal rotation, external rotation, abduction, and adduction. These data were recorded in degrees for each participant in a de-identified data sheet. In the following days, all participants began the process of being treated with the Spencer technique of the hip, bilaterally, twice weekly for four weeks, performed by members of the research team. Four weeks later, bilateral ROM measurements were retaken. These data were collected, and the authors proceeded with the analysis of the data. A summary of the timeline of this study is displayed in Figure 1.

For the two times data were collected, the degrees of flexion (F), extension (E), internal rotation (IR), external rotation (ER), abduction (AB), and adduction (AD) were measured bilaterally. These bilateral measurements were subsequently averaged together for each participant. This resulted in final data for each participant's average ROM for each movement measured at two distinct points in time: (1) initial measurements on January 18, 2024, at 5:00 PM, and (2) final measurements on February 17, 2024, at 7:00 AM.

This allowed for the determination of the percent change of each motion between the two points in time. Additionally, during these four weeks, the participants were instructed to train as they usually would while also responding to a confidential weekly survey, which retrospectively tracked the amount of distance running each participant engaged in during the four weeks and the speed at which this was done. These data allowed retrospective categorization of the participants into one of two groups: "runners" and "non-runners." "Runners" (n = 9) were defined as those who reported at least two days per week of running a sub-ten-minute mile for at least two of the four weeks. Those who did not meet this criterion (n = 11) were considered "non-runners" for this data analysis. Then, both groups had their mean percent change for each motion calculated. Analysis of this data (average percent change in ROM in the "runners" group vs. the "non-runners" group over the four weeks) was performed using 2-sample, 2-tailed t-tests. Significant findings from this analysis are summarized in the results section.

## Results

**Figure 2:**
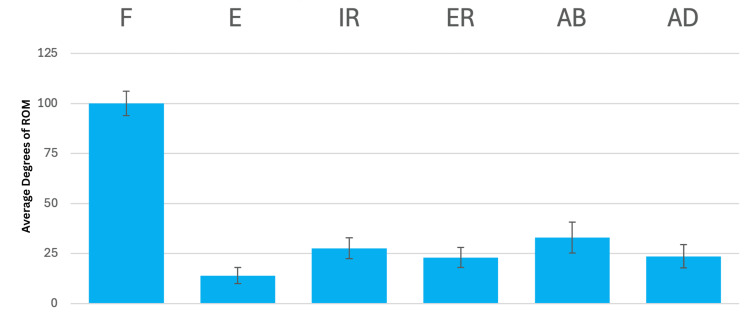
Average ROM and SD of measurements taken by the researchers on the six standard individuals Chart displaying findings of average ROM and standard deviation, in degrees, for each motion taken by members of the research team on six standard individuals. This exercise was completed to demonstrate the research team's precision of goniometric measurements prior to study onset. Error bars represent standard deviation. ROM: range of motion, SD: standard deviation, F: flexion, E: extension, IR: internal rotation, ER: external rotation, AB: abduction, AD: adduction

As mentioned in the methods, prior to original study execution, average ROM and average standard deviations for the measurements of each motion were determined using data collected by all members on seven standardized individuals [7]. Results of this investigation are displayed in Table 4 and Figure 2, demonstrating the most significant variability in terms of inter-researcher data collection for abduction (average standard deviation of 7.72°) and the least variability for extension (average standard deviation of 4.00°). Further, there were zero outliers (defined as ±3 standard deviations from the mean) in terms of measurements taken by members of the research team on the standardized individuals [7].

**Table 4:** Average ROM and SD of measurements recorded by the researchers on the six standard individuals Findings of the average ROM and standard deviation, in degrees, for each motion taken by members of the research team on six standard individuals. This information was initially published in the primary research and may also be found in O'Rorke et al. [7]. ROM: range of motion, SD: standard deviation

Motion	Average degrees of ROM	Average SD (degrees of motion)
Flexion	100.1	6.1
Extension	14.0	4.0
Internal rotation	27.7	5.2
External rotation	23.1	5.0
Abduction	33.1	7.7
Adduction	23.6	5.8

**Figure 3:**
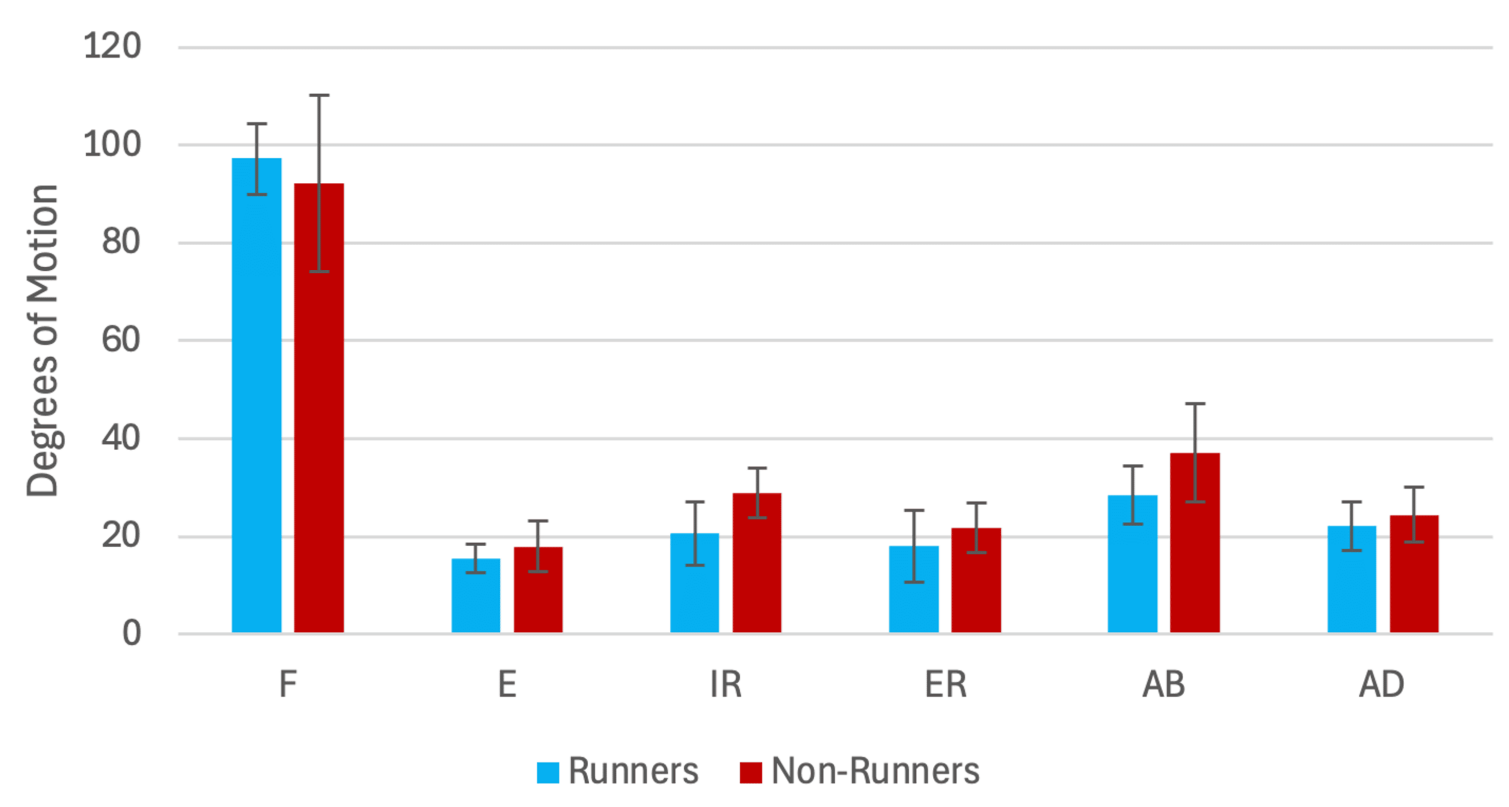
Average ROM at study onset in runners and non-runners All findings comparing runners vs non-runners in terms of average ROM at the start of the four weeks. Error lines represent the SD (degrees of motion). ROM: range of motion, SD: standard deviation, F: flexion, E: extension, IR: internal rotation, ER: external rotation, AB: abduction, AD: adduction

**Figure 4:**
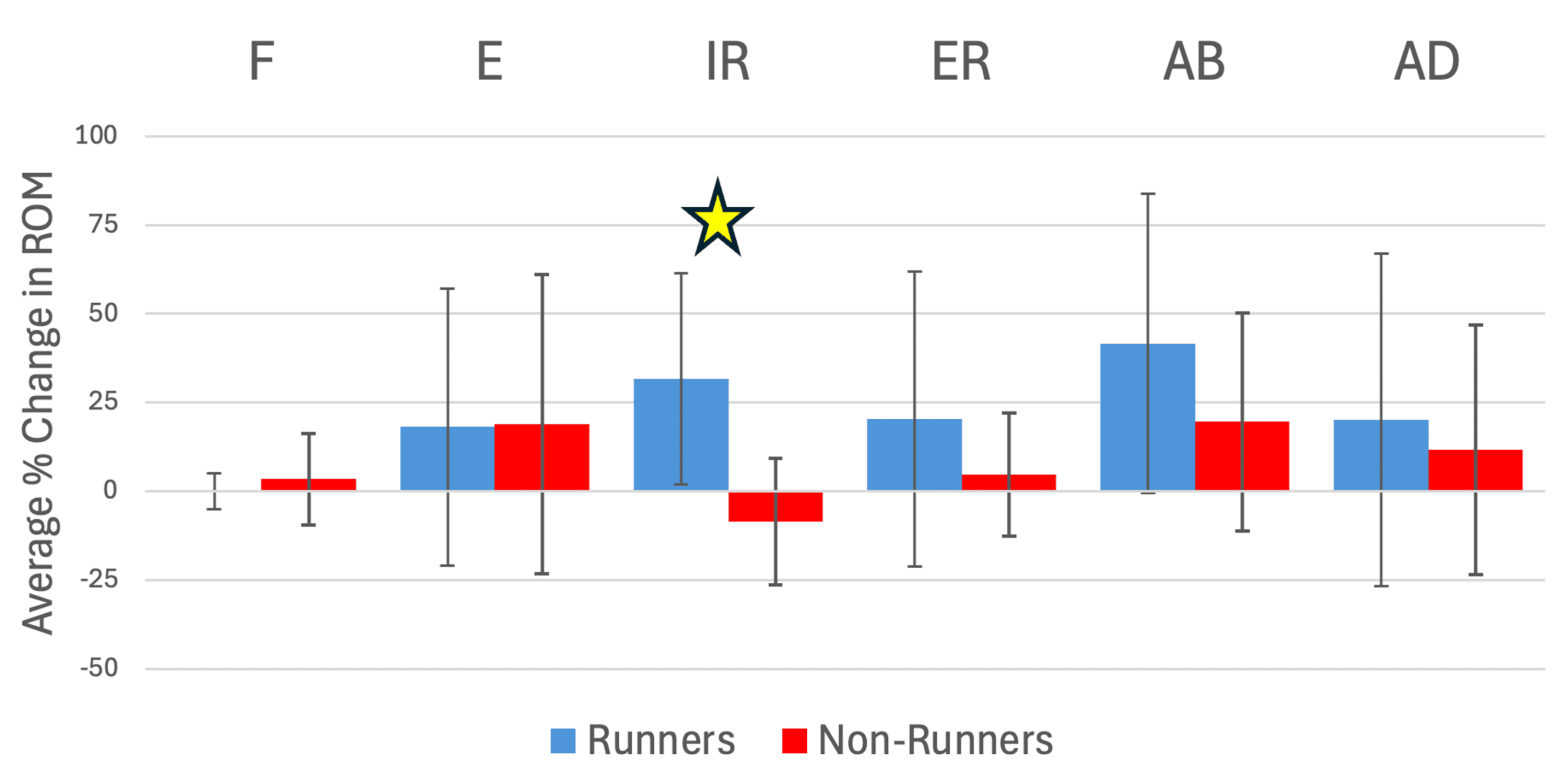
Changes in ROM over four weeks in runners and non-runners All findings comparing runners vs non-runners in terms of average percent change in ROM from start to finish of the four weeks. Error lines represent standard deviation, and the yellow star represents a statistically SD (p < 0.05). ROM: range of motion, SD: standard deviation, F: flexion, E: extension, IR: internal rotation, ER: external rotation, AB: abduction, AD: adduction

A total of 20 participants completed all the requirements of the study. The cohort had a 100% attendance rate for their twice-weekly treatment sessions as well as a 100% response rate to the exercise tracking forms. Displayed in Table 5 and Figure 3 are the ROM data at the onset of the study. Even though this study was not randomized, these findings suggest no significant difference between the groups for any motion present at study onset. The results of the analysis comparing average percent change in ROM for “runners” vs “non-runners” across the four weeks can be seen in Table 6 and Figure 4. One significant difference in average percent change in ROM for runners (n = 9) vs treatment groups (n = 11) across the four weeks was found for internal rotation, where those in the “runner” group experienced an average change of +31.7% (standard deviation of 29.8%) and the “non-runner” group experienced an average change of -8.6% (standard deviation of 17.8%) for a difference of 40.3% (95% CI: (15.40, 65.20), p = 0.001). The other findings comparing the average percent change in ROM for all other motions in “runners” vs “non-runners” produced no statistically significant difference between “runners” and “non-runners” (p > 0.05).

**Table 5:** Average ROM measurements at study onset in runners and non-runners All findings comparing runners vs non-runners in terms of average ROM at the start of the four weeks. ± SD (degrees of motion) ROM: range of motion, SD: standard deviation, CI: confidence interval

Motion	Average ROM (degrees) at study onset (runners)	Average ROM (degrees) at study onset (non-runners)	Difference (degrees of motion)	95% CI (difference in degrees ROM)	Cohen's D	P-value
Flexion	97.2 (±7.2)	92.2 (±18.0)	5.0	(-5.9, 17.5)	0.35	0.56
Extension	15.5 (±3.0)	17.9 (±5.2)	2.4	(-5.4, 13.7)	-0.55	0.62
Internal rotation	20.6 (±6.5)	28.9 (±5.1)	8.4	(-7.0, 23.2)	-1.44	0.46
External rotation	18.0 (±7.3)	21.8 (±5.0)	3.8	(-6.2, 16.8)	-0.62	0.75
Abduction	28.5 (±5.9)	37.1 (±10.0)	8.6	(-10.0, 30.0)	-1.02	0.51
Adduction	22.1 (±5.0)	24.4 (±5.6)	2.4	(-7.6, 18.3)	-0.43	0.68

**Table 6:** Changes in ROM over four weeks in runners and non-runners All findings comparing runners vs non-runners in terms of average percent change in ROM from start to finish of the four weeks. ± SD (%), * statistically significant (p < 0.05) ROM: range of motion, CI: confidence interval

Motion	Average % change in ROM (runners)	Average % change in ROM (non-runners)	Difference (%)	95% CI (difference in % change ROM)	Cohen's D	p-value
Flexion	0.1% (±5.1)	3.4% (±12.8)	3.3	(-4.8, 11.4)	-0.33	0.47
Extension	18.2% (±39.0)	19.0% (±42.1)	0.8	(-28.8, 30.4)	-0.02	0.97
Internal rotation	31.7% (±29.8)	-8.6% (±17.8)	40.3	(15.4, 65.2)	1.69	0.001*
External rotation	20.3% (±41.5)	4.7% (±17.4)	15.6	(-2.4, 33.6)	0.51	0.27
Abduction	41.6% (±42.1)	19.6% (±30.7)	22.0	(-13.0, 57.0)	0.61	0.19
Adduction	20.1% (±46.8)	11.7% (±35.1)	8.4	(-17.9, 34.7)	0.21	0.65

## Discussion

This study aimed to investigate the effects of the Spencer technique on femoroacetabular ROM in runners and non-runners over four weeks of treatment. The main finding was a significant increase in internal rotation among runners compared to non-runners (p = 0.001). No other ROM measurements showed statistically significant differences between groups. The significant difference in internal rotation between runners and non-runners could suggest that regular running affects the hip joint's response to articulatory techniques such as the Spencer technique. This may be attributable to disparities in baseline muscle tone or neuromuscular adaptations associated with running. However, the lack of significant changes amongst other motions suggests that factors beyond running status, such as individual variability in tissue response, may influence outcomes.

Several limitations must be considered, including the small sample size (n = 20), which reduces the ability to generalize findings. The four-week duration may not have been enough time to observe more significant effects, and self-reported exercise data incorporates potential recall bias. Additionally, the use of hand goniometers instead of video goniometry may have affected the accuracy of measurements. Lastly, the absence of a prescribed exercise protocol allowed for significant variation in activity outside of treatment. Regarding the validity of this study, the accuracy of femoroacetabular internal rotation measurements could have been affected by the following factors during assessment: inter-rater variability, inconsistent positioning of different patients, different forces applied by examiners, and involuntary muscle guarding. Additional external influences, such as a prior warm-up, muscle fatigue, and the time of day of measurement, could impact the recorded ROM. All of these variables have the potential to introduce measurement error, thereby decreasing the confidence in the results. To improve this study, future replications should include a longer duration of treatment, exact time-of-day measurements, a larger sample size, and more precise tools for measurement, such as video goniometry. Restricting participants' workout routines for the duration of the study could also decrease extraneous variables.

The findings of this study can be directly implemented in clinical practice, especially in sports medicine and rehabilitation. The increase in femoroacetabular internal rotation among runners following the Spencer technique demonstrates promise in improving joint mobility of individuals under repetitive stress patterns such as running. Multiple other studies have correlated restriction of internal rotation at the hip with an increased susceptibility to anterior cruciate ligament tears and intra-articular hip injuries in athletes [14,15]. Internal rotation of the stance-side hip also causes contralateral pelvic rotation that helps maintain stride efficiency and minimize excessive joint stress [16]. Considering the aforementioned benefits of increased femoroacetabular internal rotation, physicians could consider integrating the Spencer technique into their treatment plans for athletes who endure mobility restrictions, somatic dysfunctions related to running, or even those lacking significant somatic dysfunction, such as those who participated in this study. However, it is essential to consider individual responses to manipulation when determining its adequacy.

Introducing additional populations could allow researchers to assess the effects of the Spencer technique amongst different demographics. To generalize the findings of the study to different populations, including the elderly, future studies should consider implementing modifications based on the limitations of osteoarthritis or reduced physical fitness levels. Adjustments to the frequency and duration of treatment would have to be made based on the physiological differences of varying age groups, improving the universality of the study. Nevertheless, this study demonstrates that when the Spencer technique of the hip is implemented, those who engage in running regularly experience a benefit compared to those who do not run, which is valuable information for those who want to maintain joint health.

## Conclusions

This study demonstrated that using the Spencer technique of the hip as a treatment modality improves hip ROM in runners significantly more than in non-runners, specifically within internal rotation. These findings suggest an enhanced biomechanical effect of osteopathic treatments, such as the Spencer technique, when combined with recreational aerobic activity. In turn, specifically tailored OMT has the potential for greater optimization and positive neuromuscular outcomes in physically active patients. However, more research is required to explore the benefits and drawbacks of this technique across broader athletic and non-athletic populations, as it is still in its early stages as an established osteopathic treatment. Further, the generalizability of the findings of this study is strongly limited by a non-randomized, homogenous, and small sample size, requiring more expansive research to increase the applicability of these findings. Nonetheless, this research clearly displays one successful instance of the Spencer technique of improving the joint ROM and emphasizes its potential for widespread integration.
